# Has the Public Health System Provided Adequate Financial Risk Protection for Child Birth Conditions – Evidences From an Eastern Indian State

**DOI:** 10.15171/ijhpm.2018.111

**Published:** 2018-11-24

**Authors:** Sarit Kumar Rout, Sandeep Mahapatra

**Affiliations:** Indian Institute of Public Health Bhubaneswar (IIPHB), Bhubaneswar, India.

**Keywords:** Child Birth, Health Financing, Maternal Health, Odisha

## Abstract

Over the years, national and sub-national governments have introduced several initiatives to improve access to maternal and child health services in India. However, financial barriers have posed major constraints. Based upon the data of National Family Health Survey (NFHS) round 4 for Odisha state, our paper examines the out-of-pocket expenditure (OOPE) borne by households for accessing maternal and child healthcare services in a low resource setting of India. We have interpreted results of NFHS-4 by drawing inferences from literature for understanding the rising OOPE in the public health system. Findings suggests that OOPE is considerably high for maternal and child health conditions in Odisha and ranks fifth, despite the coverage of 72% women under Janani Suraksha Yojana (JSY), a condition cash transfer scheme with majority utilizing the public health system. The high OOPE on child delivery raises numerous pertinent questions about the effectiveness of the public health delivery system, and thus requires financial protection in the interest of the population that accesses public health systems in the state.

## Background

In India, healthcare is largely financed through out-of-pocket expenditure (OOPE) which stands at 63% of total health expenditure.^1^ The impoverishment that impacts high OOPE on Indian households is well established in literature. The enormous OOPE estimating between INR 32 to 39 million has driven millions of individuals to poverty.^2,3^ Further low public spending as one of the major reasons for debilitating public healthcare system of the country has been observed. Though the onus of providing affordable healthcare to its citizens rests on the public health system, most seek care in the private health sector. Though in India the public sector provides 18% of total outpatient care and 44% inpatient care,^3^ nonetheless, wide inter-state variations in utilisation pattern exists; with exception of Assam and Odisha with higher utilization in the public health system.^4^ Prominent reasons for not accessing services from public sector facilities include long waiting hours and poor quality of care among various other reasons.^4^ Moreover adding to the scenario are inadequate health workforce, insufficient drug supply, and diagnostic services, notably severe in the rural areas of the country.

Despite such constraints, over the years, India has witnessed improvements in maternal child health indicators – especially in low performing states as Chhattisgarh, Odisha, Madhya Pradesh, Jharkhand, and Rajasthan. As seen from the Sample Registration Survey, the maternal mortality ratio (MMR) has declined from 303 in 2004-2006 to 180 in 2014-2016 in Odisha, and 335 in 2004-2006 to 173 in 2014-2016 in Madhya Pradesh/Chhattisgarh.^5^ Furthermore, significant improvement in institutional delivery across the country, across states has been noted. Several evidences ascribe the improvements to the efforts undertaken by the National Health Mission (NHM).^6,7^ Among the initiatives of the NHM, the role of *Janani Suraksha Yojana* (JSY) – a conditional cash transfer programme for pregnant women from low socio-economic strata – is critical. The JSY program provides women cash incentive of US$23 (INR 1400), after child delivery in a government health facility. Since the inception of the programme about 12 years ago, the Government of India (GoI) has spent US$16.4 billion, thus increasing institutional delivery rates from 41% in 2005-2006 to 79% in 2016-2017, with more than 106 million beneficiaries.^8^ This programme has indeed helped to bring many pregnant women to the health system. However, OOPE for child birth conditions remains as a major barrier to needed healthcare. This is supported by literature from previous studies that indicate considerable proportion of Indian women faced financial hardships towards institutional delivery.^9,10^

Gopalan and Durairaj found that though a substantial increase in institutional deliveries across the country was attributed to JSY, there still existed few limitations due to high OOPE incurred by families especially for purchasing of drugs and transport.^11^

Further in order to reduce OOPE and increase healthcare access, the GoI introduced *Janani Shishu Suraksha Karyakarm* (JSSK) in 2011, which includes free treatment, drugs, diagnostics, and transport service for mother and infant in public hospitals, which is more of an entitlement scheme.^12^ Thus indicating that JSY and JSSK will help enhance access and ameliorate the persistent problem of OOPE for at least those accessing public health facilities.

However, evidences indicate that individuals are incurring OOPE even after enrolment in the scheme. Tripathi et al confirmed that a marginal decline in the OOPE from the pre-JSSK to post-JSSK, however no significant difference in catastrophic health expenditures between pre-JSSK (21.2%) and post-JSSK (15.6%) periods (*P *= .15) was seen.^13^ Another study showed that 83.5% of the study population that accessed JSSK benefits incurred OOPE. The mean expenditure calculated was INR 4289 (range: INR 150–51 200). The median OOPE was INR 1100.^14^ According to a primary survey conducted across various districts of Delhi, beneficiaries were still incurring enormous costs on health. The larger share of the expenditure was on: diagnostics that can be attributed to infrastructure bottlenecks; followed by expenditure on medicines due to lack of regular supply and availability of drugs.^15^

Even the recent National Family Health Survey (NFHS) findings highlight massive OOPE for child birth in healthcare facilities, especially higher among other states in India, fifth highest after West Bengal, Kerala, Karnataka, and Telangana.^16^ Hence bringing forth the limitation of JSSK to restrict OOPE, especially for a state like Odisha with high utilisation of public healthcare facilities (more than 70%, second highest after Assam), with an average OOPE US$62 (INR 4225) for child birth in public healthcare institution.^17^ The state has been successful in reducing infant mortality rate (IMR) from 112 per 1000 live births in 1998–1999 to 40 in 2015–2016, higher than the national average,^16,18^ despite the large share of Scheduled Tribe and scheduled caste population. Therefore, makes an apt case to investigate the high OOPE for child birth conditions in detail. In order to provide deeper understanding of the subject, this paper attempts to synthesize current evidences related to OOPE on child delivery in Odisha in context to the JSY scheme coverage as summarized in NFHS-4 and identify the probable factors affecting the expenditure. By correlating findings from our paper to similar studies, we expect to put forth valuable insights for policy implications.

## Methods

Our study draws observations from NFHS round 4 factsheets and state reports. NFHS – is a periodic survey, conducted under the stewardship of the Ministry of Health and Family Welfare (MoHFW), GoI, to provide estimates of key maternal, child and nutritional indicators at national and state levels. The total sample size for the round 4 was approximately 572 000 households in India and this is adequate to produce reliable estimates for each district and urban and rural areas. Our descriptive study examined the average OOPE for child birth in Odisha, the rising OOPE for child birth, and whether JSSK had any role in providing financial risk protection to the beneficiaries. It is important to mention here that JSSK is an entitlement scheme and anyone who accesses public health facilities qualifies to receive all provisions under this scheme. Therefore, all JSY beneficiaries if they have accessed public health facilities (reported in NFHS-4 data), are eligible to get the benefits, and are used for computation in our study.

## Results

**Figure 1:**
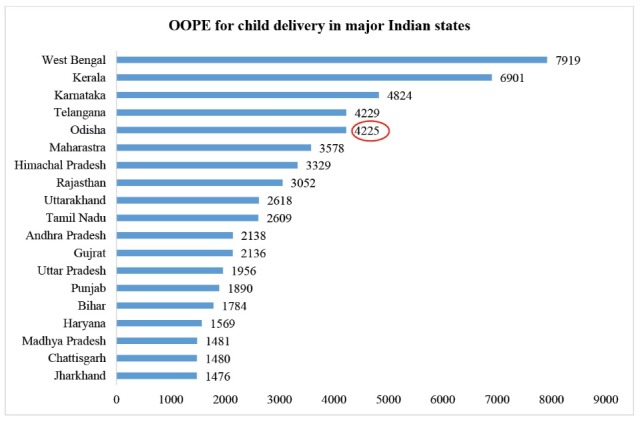
OOPE (in Rupee) Expenditure in Major Indian States. Abbreviation: OOPE, out-of-pocket expenditure.

The findings of NFHS-4 suggest that OOPE on child birth in public health facility is relatively high in Odisha. As presented in Figure 1, the average OOPE for child birth in public health facility is US$62 (INR 4225), and on comparison to other states, it ranks fifth after West Bengal, Kerala, Karnataka, and Telangana.

**Figure 2:**
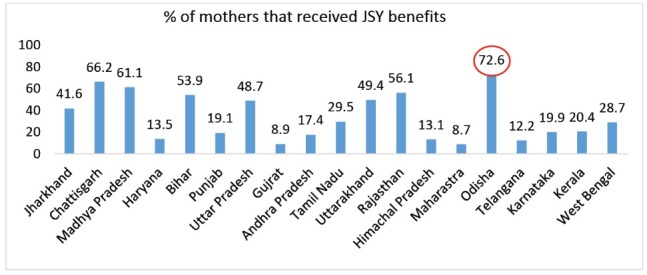
State Wise JSY Beneficiaries in India. Abbreviation: JSY, Janani Suraksha Yojana.

The percentage of mothers that received JSY benefits was highest in Odisha followed by Chhattisgarh and Madhya Pradesh (Figure 2). Hence, it is assumed that the scheme has been successful in bringing more women to deliver in public healthcare institutions.

**Figure 3:**
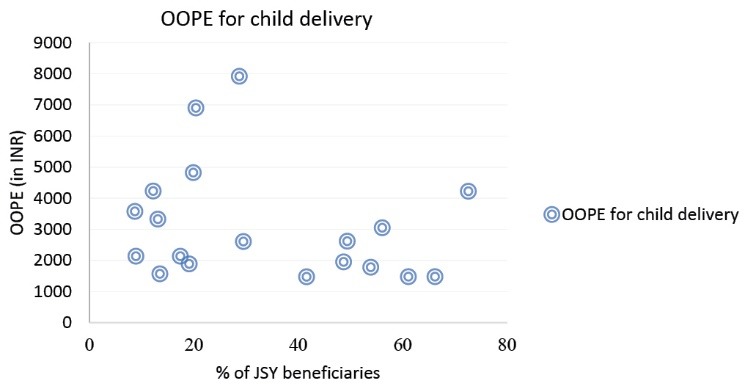
JSY Beneficiaries (%) and OOPE (in INR) for Child Delivery. Abbreviations: JSY, Janani Suraksha Yojana; OOPE, out-of-pocket expenditure.

Further as presented in the scattered plot of Figure 3, an inverse relationship between percentage of JSY beneficiaries and the OOPE for child delivery was noted. However, an exception is noticed for Odisha where the OOPE is high despite of high percentage of women receiving financial assistance under JSY. Hence this requires to be examined carefully especially the effectiveness of the scheme and the functioning of the public health system in the state.

Another indicator that needs to be highlighted in this context is the percentage of institutional delivery in public health facilities in the state. In comparison to the major states, Odisha accounts for the highest (around 75%) institutional delivery in the public health facilities. However, despite the majority utilizing public health facilities for child delivery, high OOPE for child birth conditions is quite disquieting.

## Discussion

Our paper presents results from preliminary findings of NFHS-4 along with other available evidences to provide a scientific concoction of insights on the high OOPE for child birth conditions in Odisha, especially where utilization of public health facilities is high.

Over the years, the public healthcare system of Odisha, has been successful in increasing institutional deliveries from 39% in 2005-2006 to 76% in 2015-2016.^16,17^ Additionally, this improvement in institutional deliveries has been seen across the districts of the state including remote, tribal districts. Though the striking success of JSY scheme in the state also contributes to the positive growth, this success is undermined since the public health system fails to provide financial risk protection to the beneficiaries. In Odisha even though more than 70% inpatient and outpatient visits are in public health facilities, households still incur an average OOPE of INR 4225 for child delivery, as evident from the findings of NFHS-4 and other studies conducted in the state.^19,20^ A study conducted on JSY in districts of Odisha showed that the incentive induced out-of-pocket spending for mothers and was unable to address maternal care requirements comprehensively.^11^ The findings from another study conducted in urban slums of the capital, Bhubaneswar suggested that JSSK scheme which was launched to reduce OOPE on child birth to zero, was also not successful in reducing the same.^21^ However, in the absence of schemes like JSY and JSSK situation in the state would have been worse.

According to the State Health Accounts report – Odisha, the share of household expenditure on health was 76.3%, with more than 58% incurred on drugs, 12% on diagnostics, and 8.9% on transportation.^22^ Further evidences also illustrate increase in OOPE due to non-availability of medicines in the state. Sharma and Bothra noted that more than half of the OOPEs incurred during hospitalisation was due to medicines.^23^ Additionally, a cross-sectional survey in 2008 found that the average amount spent by JSY beneficiaries on medicines and other services ranged from INR 299 in Madhya Pradesh to INR 1638 in Odisha.^19,24^ High OOPE could be attributed to caesarean sections, but counter intuitively, as per NFHS-4, only 12% such procedures were accounted for in public health facilities of Odisha. In the light of available evidences, inadequate financial coverage by JSY scheme, additional expenses incurred by mothers on medicines, baby food and informal payments have been reported to be the contributing factors to the overall increase in OOPE.^11,25^ Furthermore, studies also bring to the forefront that although the JSY has undeniably increased institutional delivery significantly, the poorest and the least educated women may not always be the ones to receive the benefit, and, hence there is a pressing need to target the poorest women.^26^

The Odisha state government has strived to introduce several innovative schemes to reduce financial hardships for individuals using public health services. For instance, the *Niramaya* scheme^27,28^ provides free drugs at all levels of care, along with the conditional cash delivery schemes – *Mamata*^29^ for providing cash incentives to pregnant and lactating mothers, and JSSK.^30^ for providing free treatment and diagnostic services for child birth conditions.

However, the high OOPE in public health facilities for child birth conditions in Odisha despite implementation of several financial assistance schemes raises serious questions regarding effectiveness of the schemes to provide financial risk protection to the poor households. Hence in this context, it is vital to identify the gaps and suggest the appropriate strategies to improve the health delivery system. We acknowledge certain limitations of the study. Data used is primarily from the factsheets which may have restricted the understanding of certain issues. However, considering the length restrictions of the paper, we believe the study puts forth important insight on OOPE associated with mother and child services in one of the poorer states of the country. Thus, encourages further investigation of the subject in detail that may provide insights to the policy makers to design innovative schemes.

## Conclusion

Odisha has made important strides in addressing maternal and child health problems. However, synthesis of evidences from NHFS-4 and other literature show that despite the high reliance on public health facilities, the higher OOPE for child birth conditions is a major area of concern This requires evaluation of the effectiveness of various demand side financing schemes including JSSK, which is a free entitlement scheme in public health facilities. Strengthening these schemes is imperative for protecting the interest of the people who mainly utilise public health system in the state.

## Ethical issues

Not applicable.

## Competing interests

Authors declare that they have no competing interests.

## Authors’ contributions

SKR conceptualized the paper. Both authors were involved in the data analysis and interpretation. SM was involved in drafting the paper. SKR revised the draft and critically analyzed the paper. Both the authors have approved the final version of the article submitted.
